# The genome sequence of the True Lover's Knot moth, *Lycophotia porphyrea *(Denis & Schiffermüller), 1775

**DOI:** 10.12688/wellcomeopenres.22897.2

**Published:** 2025-03-18

**Authors:** Jonathan Davis, Andy Griffiths, Wellcome Sanger Institute Tree of Life Management, Samples and Laboratory team, Wellcome Sanger Institute Scientific Operations: Sequencing Operations, Wellcome Sanger Institute Tree of Life Core Informatics team, Tree of Life Core Informatics collective, Darwin Tree of Life Consortium

**Affiliations:** 1 Independent researcher, Lanarck, Scotland, UK; 2 Wellcome Sanger Institute, Hinxton, England, UK; 3 Royal Botanic Garden Edinburgh, Edinburgh, Scotland, UK

**Keywords:** Lycophotia porphyrea, True Lover's Knot moth, genome sequence, chromosomal, Lepidoptera

## Abstract

We present a genome assembly from an individual male *Lycophotia porphyrea* (the True Lover’s Knot; Arthropoda; Insecta; Lepidoptera; Noctuidae). The genome sequence has a total length of 542.40 megabases. Most of the assembly is scaffolded into 31 chromosomal pseudomolecules, including the Z sex chromosome. The mitochondrial genome has also been assembled and is 15.39 kilobases in length. Gene annotation of this assembly at Ensembl identified 17,907 protein-coding genes.

## Species taxonomy

Eukaryota; Opisthokonta; Metazoa; Eumetazoa; Bilateria; Protostomia; Ecdysozoa; Panarthropoda; Arthropoda; Mandibulata; Pancrustacea; Hexapoda; Insecta; Dicondylia; Pterygota; Neoptera; Endopterygota; Amphiesmenoptera; Lepidoptera; Glossata; Neolepidoptera; Heteroneura; Ditrysia; Obtectomera; Noctuoidea; Noctuidae; Noctuinae; Noctuini; *Lycophotia*; *Lycophotia porphyria* (Denis & Schiffermüller), 1775 (NCBI:txid987975).

## Background

**Figure 1.**
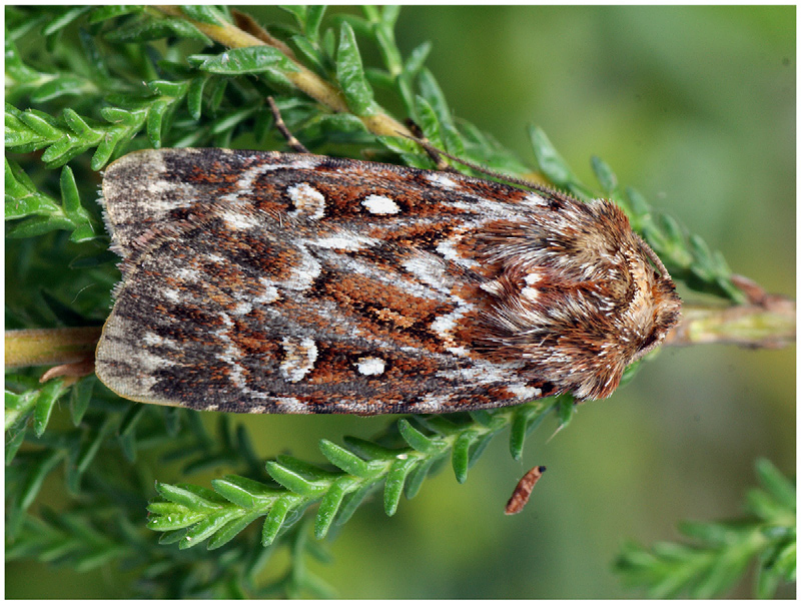
Image of *Lycophotia porphyrea* (not the specimen used for genome sequencing). Photograph by Rudolphous.

*Lycophotia porphyria* (True Lover’s Knot) ( Figure 1) is a macromoth of the Noctuidae family. It is distributed primarily across the west Palaearctic, and it is commonly found in the northern and western parts of Europe, with significant occurrences recorded in countries including United Kingdom, Germany, France, and the Scandinavian countries ( GBIF Secretariat, 2024). It is also observed in parts of eastern Europe, including Poland and the Baltic states ( GBIF Secretariat, 2024). It occurs throughout Britain, specifically in moorland habitats where its larval foodplant heather (both *Calluna vulgaris* and *Erica cinerea*) is found. It is also known to feed on cultivated heathers. In Britain, its distribution and abundance has declined recently ( Randle *et al.*, 2019), though records in Scotland suggest this change has not been observed there ( Leverton & Cubitt, 2024).

*Lycophotia porphyria* overwinters as a fully grown larva, before pupating in the ground. The adult moth is on the wing between June and August ( Waring *et al.*, 2017).

Its cryptic markings camouflage it perfectly when it is settled in heather. The forewing pattern is said to resemble a knot with a double loop. In antiquity, knots of various kinds were associated with affection or love, which is the origin of this moth’s vernacular name ( Marren, 2019).

Here we present a chromosomally complete genome sequence for *Lycophotia porphyria*, based on a male specimen from Little Sparta, a garden in South Lanarkshire, Scotland, UK.

## Genome sequence report

The genome of an adult male *Lycophotia porphyrea* was sequenced using Pacific Biosciences single-molecule HiFi long reads, generating a total of 27.60 Gb (gigabases) from 2.75 million reads, providing approximately 49-fold coverage. Primary assembly contigs were scaffolded with chromosome conformation Hi-C data, which produced 157.18 Gb from 1,040.90 million reads, yielding an approximate coverage of 290-fold. Specimen and sequencing information is summarised in Table 1.

**Table 1.** Specimen and sequencing data for *Lycophotia porphyrea*.

Project information			
**Study title**	Lycophotia porphyrea (true lover's knot)		
**Umbrella BioProject**	PRJEB61365		
**Species**	*Lycophotia porphyrea*		
**BioSample**	SAMEA112198378		
**NCBI taxonomy ID**	987975		
Specimen information			
**Technology**	**ToLID**	**BioSample accession**	**Organism part**
**PacBio long read sequencing**	ilLycPorp1	SAMEA112198441	thorax
**Hi-C sequencing**	ilLycPorp1	SAMEA112198441	thorax
**RNA sequencing**	ilLycPorp2	SAMEA112360824	abdomen
Sequencing information			
**Platform**	**Run accession**	**Read count**	**Base count (Gb)**
**Hi-C Illumina NovaSeq 6000**	ERR11242566	1.04e+09	157.18
**PacBio Sequel IIe**	ERR11242142	2.75e+06	27.6
**RNA Illumina NovaSeq 6000**	ERR12035189	5.70e+07	8.61

**Figure 2.**
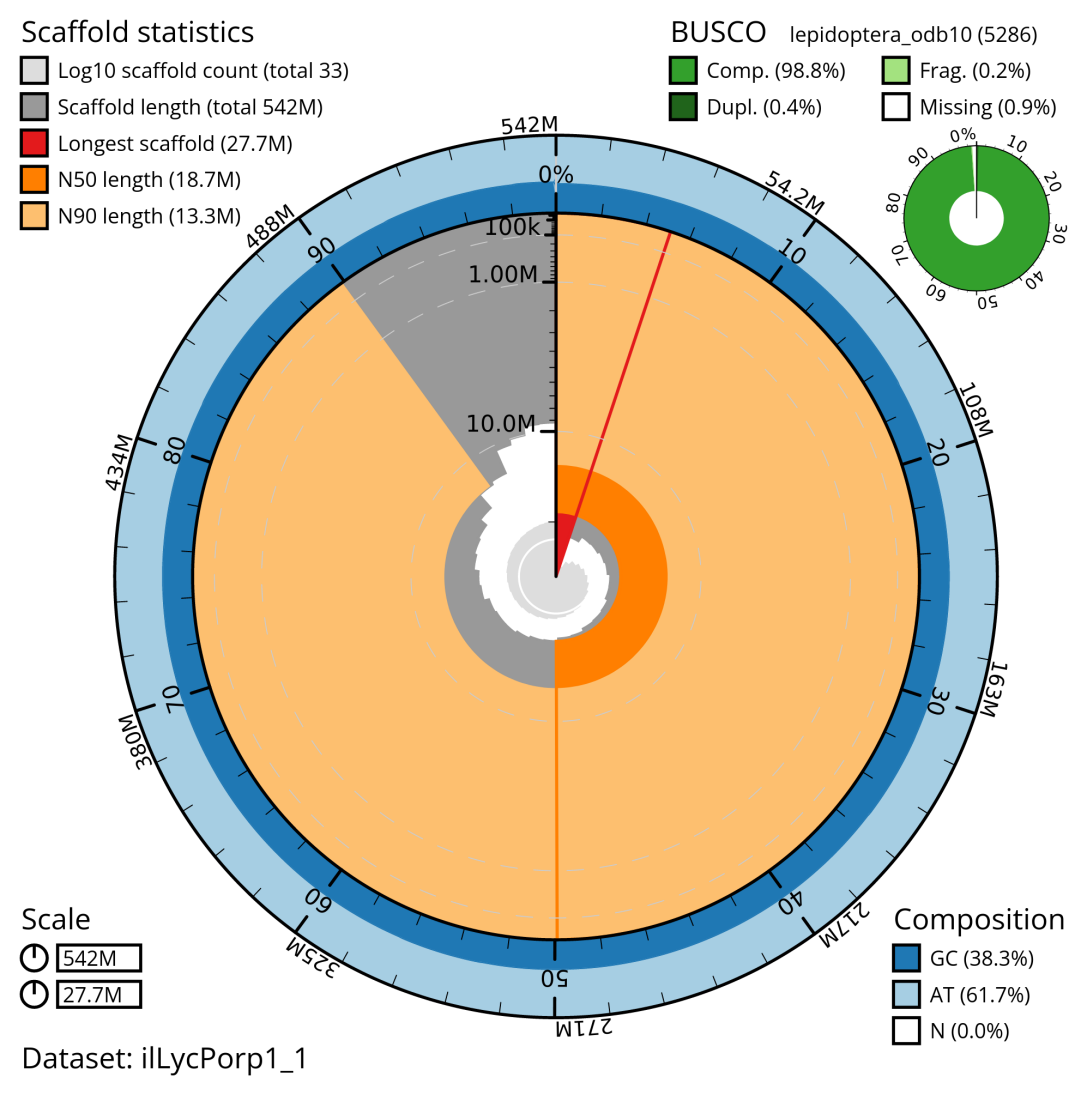
Genome assembly of *Lycophotia porphyrea*, ilLycPorp1.1: metrics. The BlobToolKit snail plot shows N50 metrics and BUSCO gene completeness. The main plot is divided into 1,000 size-ordered bins around the circumference with each bin representing 0.1% of the 542,420,237 bp assembly. The distribution of scaffold lengths is shown in dark grey with the plot radius scaled to the longest scaffold present in the assembly (27,685,435 bp, shown in red). Orange and pale-orange arcs show the N50 and N90 scaffold lengths (18,690,029 and 13,292,147 bp), respectively. The pale grey spiral shows the cumulative scaffold count on a log scale with white scale lines showing successive orders of magnitude. The blue and pale-blue area around the outside of the plot shows the distribution of GC, AT and N percentages in the same bins as the inner plot. A summary of complete, fragmented, duplicated and missing BUSCO genes in the lepidoptera_odb10 set is shown in the top right. An interactive version of this figure is available at https://blobtoolkit.genomehubs.org/view/ilLycPorp1_1/dataset/ilLycPorp1_1/snail.

**Figure 3.**
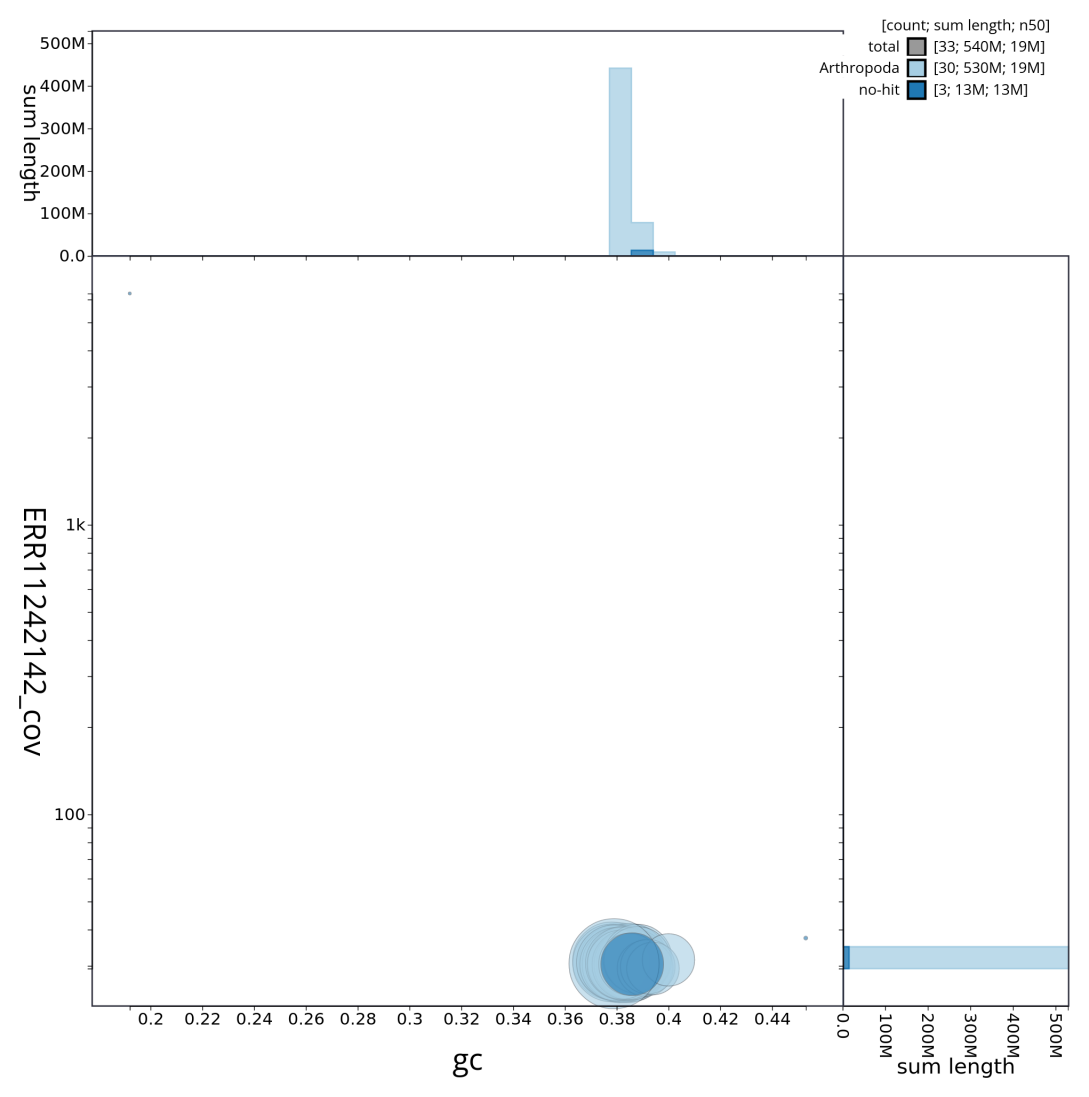
Genome assembly of *Lycophotia porphyrea*, ilLycPorp1.1: BlobToolKit GC-coverage plot. Sequences are coloured by phylum. Circles are sized in proportion to sequence length. Histograms show the distribution of sequence length sum along each axis. An interactive version of this figure is available at https://blobtoolkit.genomehubs.org/view/ilLycPorp1_1/dataset/ilLycPorp1_1/blob.

**Figure 4.**
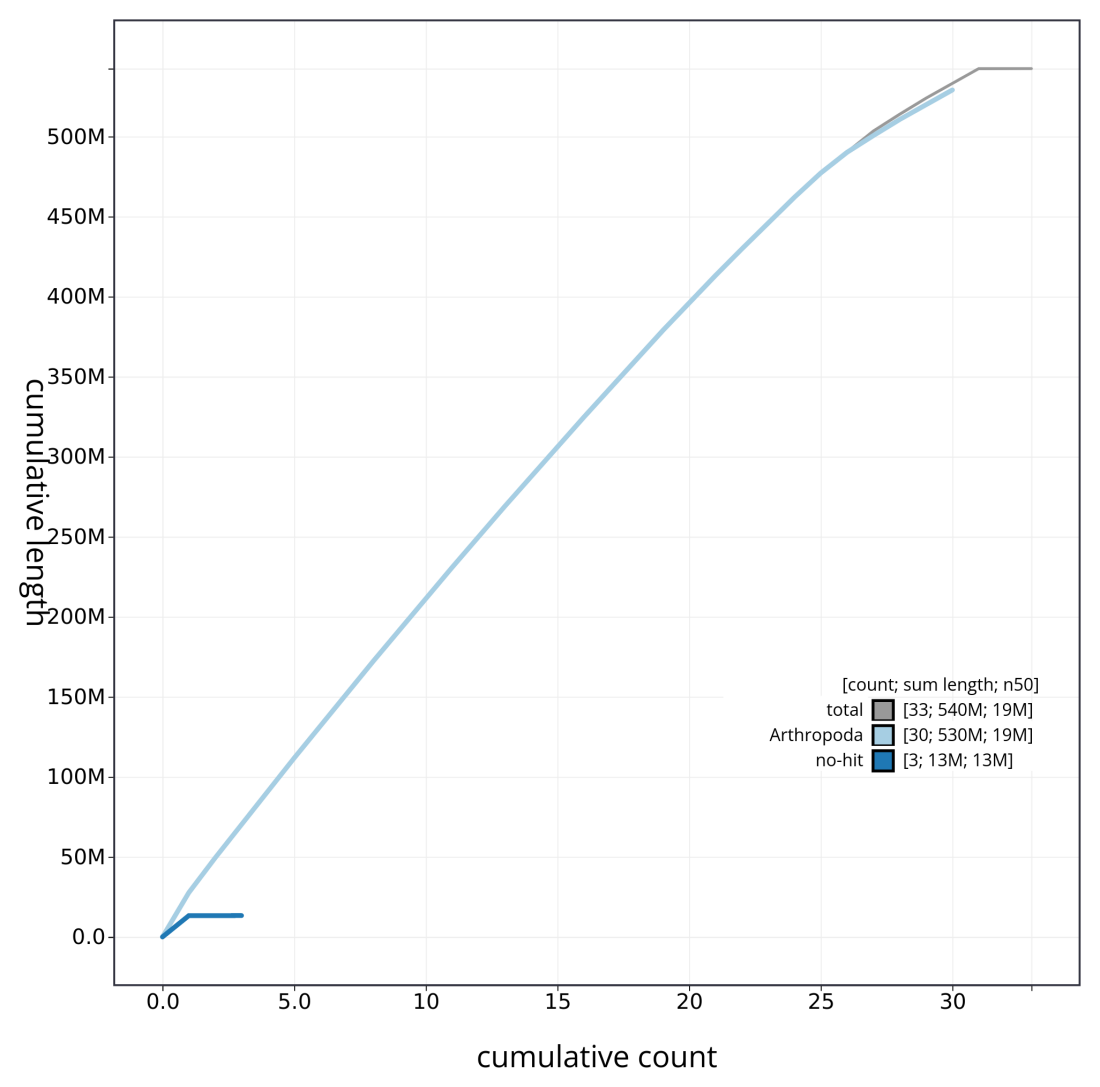
Genome assembly of *Lycophotia porphyrea* ilLycPorp1.1: BlobToolKit cumulative sequence plot. The grey line shows cumulative length for all sequences. Coloured lines show cumulative lengths of sequences assigned to each phylum using the buscogenes taxrule. An interactive version of this figure is available at https://blobtoolkit.genomehubs.org/view/ilLycPorp1_1/dataset/ilLycPorp1_1/cumulative.

**Figure 5.**
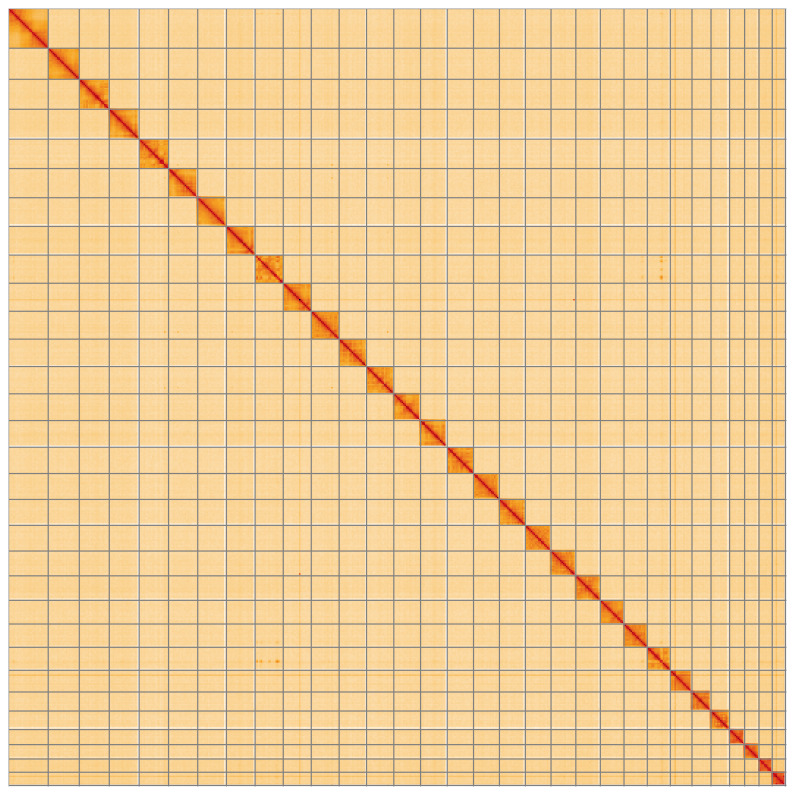
Genome assembly of *Lycophotia porphyrea* ilLycPorp1.1: Hi-C contact map of the ilLycPorp1.1 assembly, visualised using HiGlass. Chromosomes are shown in order of size from left to right and top to bottom. An interactive version of this figure may be viewed at https://genome-note-higlass.tol.sanger.ac.uk/l/?d=RFuqWxYsTyy2euv4L7RNug.

Manual assembly curation corrected 10 missing joins or mis-joins and two haplotypic duplications, reducing the scaffold number by 10.81%. The final assembly has a total length of 542.40 Mb in 32 sequence scaffolds, with 54 gaps. The scaffold N50 is18.7 Mb ( Table 2). The snail plot in Figure 2 provides a summary of the assembly statistics, while the distribution of assembly scaffolds on GC proportion and coverage is shown in Figure 3. The cumulative assembly plot in Figure 4 shows curves for subsets of scaffolds assigned to different phyla. Most (99.99%) of the assembly sequence was assigned to 31 chromosomal-level scaffolds, representing 30 autosomes and the Z sex chromosome. Chromosome-scale scaffolds confirmed by the Hi-C data are named in order of size ( Figure 5; Table 3). The Z chromosome was identified based on synteny with *Xestia c-nigrum* (GCA_916618015.1) ( Broad *et al.*, 2022). While not fully phased, the assembly deposited is of one haplotype. Contigs corresponding to the second haplotype have also been deposited. The mitochondrial genome was also assembled and can be found as a contig within the multifasta file of the genome submission.

**Table 2.** Genome assembly data for *Lycophotia porphyrea*, ilLycPorp1.1.

Genome assembly		
Assembly name	ilLycPorp1.1	
Assembly accession	GCA_950005105.1	
*Accession of alternate haplotype*	*GCA_950005095.1*	
Span (Mb)	542.40	
Number of contigs	87	
Contig N50 length (Mb)	11.1	
Number of scaffolds	32	
Scaffold N50 length (Mb)	18.7	
Longest scaffold (Mb)	27.69	
Assembly metrics *		*Benchmark*
Consensus quality (QV)	69.1	*≥ 40*
*k*-mer completeness	Primary: 80.30%, alternate: 80.23%, combined: 99.63%	*≥ 95%*
BUSCO **	C:98.8%[S:98.4%,D:0.4%], F:0.2%,M:0.9%,n:5,286	*C ≥ 95%*
Percentage of assembly mapped to chromosomes	99.99%	*≥ 90%*
Sex chromosomes	Z	*localised homologous pairs*
Organelles	Mitochondrial genome: 15.39 kb	*complete single alleles*
Genome annotation of assembly GCA_950005105.1 at Ensembl		
Number of protein-coding genes	17,907	
Number of gene transcripts	18,090	

* Assembly metric benchmarks are adapted from column VGP-2020 of “Table 1: Proposed standards and metrics for defining genome assembly quality” from Rhie *et al.* (2021). ** BUSCO scores based on the lepidoptera_odb10 BUSCO set using version 5.3.2. C = complete [S = single copy, D = duplicated], F = fragmented, M = missing, n = number of orthologues in comparison. A full set of BUSCO scores is available at https://blobtoolkit.genomehubs.org/view/ilLycPorp1_1/dataset/ilLycPorp1_1/busco.

**Table 3.** Chromosomal pseudomolecules in the genome assembly of *Lycophotia porphyrea*, ilLycPorp1.

INSDC accession	Name	Length (Mb)	GC%
OX465384.1	1	21.69	38.0
OX465385.1	2	20.93	38.5
OX465386.1	3	20.84	38.5
OX465387.1	4	20.54	38.0
OX465388.1	5	20.37	38.0
OX465389.1	6	20.03	38.0
OX465390.1	7	20.01	38.5
OX465391.1	8	19.67	38.5
OX465392.1	9	19.56	38.0
OX465393.1	10	19.47	38.0
OX465394.1	11	19.12	38.0
OX465395.1	12	19.04	38.0
OX465396.1	13	18.69	38.0
OX465397.1	14	18.61	38.5
OX465398.1	15	18.36	38.0
OX465399.1	16	18.09	38.5
OX465400.1	17	18.09	38.5
OX465401.1	18	17.94	38.0
OX465402.1	19	17.31	38.5
OX465403.1	20	17.11	38.5
OX465404.1	21	16.53	38.5
OX465405.1	22	16.26	38.0
OX465406.1	23	16.06	39.0
OX465407.1	24	15.14	39.0
OX465408.1	25	13.29	38.5
OX465409.1	26	12.95	38.5
OX465410.1	27	10.51	39.0
OX465411.1	28	10.03	39.0
OX465412.1	29	9.25	39.5
OX465413.1	30	9.2	40.0
OX465383.1	Z	27.69	38.0
OX465414.1	MT	0.02	19.5

The estimated Quality Value (QV) of the final assembly is 69.1 with *k*-mer completeness of 99.63% for the combined assemblies. The assembly has a BUSCO v5.3.2 completeness of 98.8% (single = 98.4%, duplicated = 0.4%), using the lepidoptera_odb10 reference set ( *n* = 5,286).

Metadata for specimens, BOLD barcode results, spectra estimates, sequencing runs, contaminants and pre-curation assembly statistics are given at https://links.tol.sanger.ac.uk/species/987975.

## Genome annotation report

The *Lycophotia porphyrea* genome assembly (GCA_950005105.1) was annotated at the European Bioinformatics Institute (EBI) on Ensembl Rapid Release. The resulting annotation includes 18,090 transcribed mRNAs from 17,907 protein-coding genes ( Table 2; https://rapid.ensembl.org/Lycophotia_porphyrea_GCA_950005105.1/Info/Index). The average transcript length is 7,648.61. There are 1.01 coding transcripts per gene and 5.58 exons per transcript.

## Methods

### Sample acquisition and nucleic acid extraction

An adult male *Lycophotia porphyrea* (specimen ID SAN00002571, ToLID ilLycPorp1) was collected from Little Sparta, South Lanarkshire, Scotland, UK (latitude 55.72, longitude –3.51) on 2022-06-17 using a moth trap. The specimen was collected and identified by Jo Davis (independent researcher) and preserved by flash-freezing.

The specimen used for RNA sequencing (specimen ID SAN00002581, ToLID ilLycPorp2) was an adult specimen collected from Carrifran Wildwood, Scotland (latitude 55.41, longitude –3.34) on 2022-06-23. The specimen was collected and identified by Andy Griffiths (Sanger) and preserved on dry ice.

The workflow for high molecular weight (HMW) DNA extraction at the Wellcome Sanger Institute (WSI) Tree of Life Core Laboratory includes a sequence of core procedures: sample preparation; sample homogenisation, DNA extraction, fragmentation, and clean-up. In sample preparation, the ilLycPorp1 sample was weighed and dissected on dry ice ( Jay *et al.*, 2023). Tissue from thorax was homogenised using a PowerMasher II tissue disruptor ( Denton *et al.*, 2023a).

HMW DNA was extracted in the WSI Scientific Operations core using the Automated MagAttract v2 protocol ( Oatley *et al.*, 2023). The DNA was sheared into an average fragment size of 12–20 kb in a Megaruptor 3 system ( Bates *et al.*, 2023). Sheared DNA was purified by solid-phase reversible immobilisation, using AMPure PB beads to eliminate shorter fragments and concentrate the DNA ( Strickland *et al.*, 2023). The concentration of the sheared and purified DNA was assessed using a Nanodrop spectrophotometer and Qubit Fluorometer using the Qubit dsDNA High Sensitivity Assay kit. Fragment size distribution was evaluated by running the sample on the FemtoPulse system.

RNA was extracted from tissue of ilLycPorp2 in the Tree of Life Laboratory at the WSI using the RNA Extraction: Automated MagMax™ *mir*Vana protocol ( do Amaral *et al.*, 2023). The RNA concentration was assessed using a Nanodrop spectrophotometer and a Qubit Fluorometer using the Qubit RNA Broad-Range Assay kit. Analysis of the integrity of the RNA was done using the Agilent RNA 6000 Pico Kit and Eukaryotic Total RNA assay.

Protocols developed by the WSI Tree of Life laboratory are publicly available on protocols.io ( Denton *et al.*, 2023b).

### Sequencing

Pacific Biosciences HiFi circular consensus DNA sequencing libraries were constructed according to the manufacturers’ instructions. Poly(A) RNA-Seq libraries were constructed using the NEB Ultra II RNA Library Prep kit. DNA and RNA sequencing was performed by the Scientific Operations core at the WSI on Pacific Biosciences Sequel IIe (HiFi) and Illumina NovaSeq 6000 (RNA-Seq) instruments. Hi-C data were also generated from thorax tissue of ilLycPorp1 using the Arima-HiC v2 kit. The Hi-C sequencing was performed using paired-end sequencing with a read length of 150 bp on the Illumina NovaSeq 6000 instrument.

### Genome assembly, curation and evaluation

***Assembly***

The HiFi reads were first assembled using Hifiasm ( Cheng *et al.*, 2021) with the --primary option. Haplotypic duplications were identified and removed using purge_dups ( Guan *et al.*, 2020). The Hi-C reads were mapped to the primary contigs using bwa-mem2 ( Vasimuddin *et al.*, 2019). The contigs were further scaffolded using the provided Hi-C data ( Rao *et al.*, 2014) in YaHS ( Zhou *et al.*, 2023) using the --break option. The scaffolded assemblies were evaluated using Gfastats ( Formenti *et al.*, 2022), BUSCO ( Manni *et al.*, 2021) and Merqury.FK ( Rhie *et al.*, 2020).

The mitochondrial genome was assembled using MitoHiFi ( Uliano-Silva *et al.*, 2023), which runs MitoFinder ( Allio *et al.*, 2020) and uses these annotations to select the final mitochondrial contig and to ensure the general quality of the sequence.

***Assembly curation***

The assembly was decontaminated using the Assembly Screen for Cobionts and Contaminants (ASCC) pipeline (article in preparation). Manual curation was primarily conducted using PretextView ( Harry, 2022), with additional insights provided by JBrowse2 ( Diesh *et al.*, 2023) and HiGlass ( Kerpedjiev *et al.*, 2018). Scaffolds were visually inspected and corrected as described by Howe *et al*. (2021). Any identified contamination, missed joins, and mis-joins were corrected, and duplicate sequences were tagged and removed. Sex chromosomes were identified by synteny analysis. The entire process is documented at https://gitlab.com/wtsi-grit/rapid-curation (article in preparation).

***Evaluation of the final assembly***

A Hi-C map for the final assembly was produced using bwa-mem2 ( Vasimuddin *et al.*, 2019) in the Cooler file format ( Abdennur & Mirny, 2020). To assess the assembly metrics, the *k*-mer completeness and QV consensus quality values were calculated in Merqury.FK ( Rhie *et al.*, 2020). This work was done using Nextflow ( Di Tommaso *et al.*, 2017) DSL2 pipelines. The genome was analysed within the BlobToolKit environment ( Challis *et al.*, 2020) and BUSCO scores ( Manni *et al.*, 2021; Simão *et al.*, 2015) were calculated.

The genome evaluation pipelines were developed using the nf-core tooling ( Ewels *et al.*, 2020), use MultiQC ( Ewels *et al.*, 2016), and make extensive use of the Conda package manager, the Bioconda initiative ( Grüning *et al.*, 2018), the Biocontainers infrastructure ( da Veiga Leprevost *et al.*, 2017), and the Docker ( Merkel, 2014) and Singularity ( Kurtzer *et al.*, 2017) containerisation solutions.

Table 4 contains a list of relevant software tool versions and sources.

**Table 4.** Software tools: versions and sources.

Software tool	Version	Source
BlobToolKit	4.2.1	https://github.com/blobtoolkit/blobtoolkit
BUSCO	5.3.2	https://gitlab.com/ezlab/busco
bwa-mem2	2.2.1	https://github.com/bwa-mem2/bwa-mem2
Gfastats	1.3.6	https://github.com/vgl-hub/gfastats
Hifiasm	0.16.1-r375	https://github.com/chhylp123/hifiasm
HiGlass	1.11.6	https://github.com/higlass/higlass
Merqury.FK	d00d98157618f4e8d1a9 190026b19b471055b22e	https://github.com/thegenemyers/MERQURY.FK
MitoHiFi	2	https://github.com/marcelauliano/MitoHiFi
PretextView	0.2.5	https://github.com/wtsi-hpag/PretextView
purge_dups	1.2.3	https://github.com/dfguan/purge_dups
YaHS	yahs-1.1.91eebc2	https://github.com/c-zhou/yahs

### Genome annotation

The BRAKER2 pipeline ( Brůna *et al.*, 2021) was used in the default protein mode to generate annotation for the *Lycophotia porphyrea* assembly (GCA_950005105.1) in Ensembl Rapid Release at the EBI.

### Wellcome Sanger Institute – Legal and Governance

The materials that have contributed to this genome note have been supplied by a Darwin Tree of Life Partner. The submission of materials by a Darwin Tree of Life Partner is subject to the **‘Darwin Tree of Life Project Sampling Code of Practice’**, which can be found in full on the Darwin Tree of Life website here. By agreeing with and signing up to the Sampling Code of Practice, the Darwin Tree of Life Partner agrees they will meet the legal and ethical requirements and standards set out within this document in respect of all samples acquired for, and supplied to, the Darwin Tree of Life Project.

Further, the Wellcome Sanger Institute employs a process whereby due diligence is carried out proportionate to the nature of the materials themselves, and the circumstances under which they have been/are to be collected and provided for use. The purpose of this is to address and mitigate any potential legal and/or ethical implications of receipt and use of the materials as part of the research project, and to ensure that in doing so we align with best practice wherever possible. The overarching areas of consideration are:

•   Ethical review of provenance and sourcing of the material

•   Legality of collection, transfer and use (national and international)

Each transfer of samples is further undertaken according to a Research Collaboration Agreement or Material Transfer Agreement entered into by the Darwin Tree of Life Partner, Genome Research Limited (operating as the Wellcome Sanger Institute), and in some circumstances other Darwin Tree of Life collaborators.

## Data Availability

European Nucleotide Archive: *Lycophotia porphyrea* (true lover's knot). Accession number PRJEB61365; https://identifiers.org/ena.embl/PRJEB61365. The genome sequence is released openly for reuse. The *Lycophotia porphyrea* genome sequencing initiative is part of the Darwin Tree of Life (DToL) project. All raw sequence data and the assembly have been deposited in INSDC databases. Raw data and assembly accession identifiers are reported in Table 1 and Table 2.

## Author information

Members of the Wellcome Sanger Institute Tree of Life Management, Samples and Laboratory team are listed here: https://doi.org/10.5281/zenodo.12162482.

Members of Wellcome Sanger Institute Scientific Operations: Sequencing Operations are listed here: https://doi.org/10.5281/zenodo.12165051.

Members of the Wellcome Sanger Institute Tree of Life Core Informatics team are listed here: https://doi.org/10.5281/zenodo.12160324.

Members of the Tree of Life Core Informatics collective are listed here: https://doi.org/10.5281/zenodo.5013541.

Members of the Darwin Tree of Life Consortium are listed here: https://doi.org/10.5281/zenodo.4783558.
