# Supplementary material for: The genome sequence of the True Lover's Knot moth, Lycophotia porphyrea (Denis & Schiffermüller), 1775
Source: Wellcome Open Res. 2025 Mar 18;9:469. Originally published 2024 Aug 13. [Version 2] doi: 10.12688/wellcomeopenres.22897.2 (PMC11923537; doi:10.12688/wellcomeopenres.22897.2)
Supplement: Peer review history

# Reviewer report: Reviewer response for version 2

**DOI:** 10.21956/wellcomeopenres.26433.r120809

**Published:** 2025-03-19

**Authors:** Arun Arumugaperumal

**Affiliations:** 1 Department of Biotechnology, Rajalakshmi Engineering College, Thandalam 602105, Chennai, Tamil Nadu, India

The suggested corrections in the main manuscript has been carried out. The data note can now be indexed.

Are sufficient details of methods and materials provided to allow replication by others?

Yes

Is the rationale for creating the dataset(s) clearly described?

Yes

Are the datasets clearly presented in a useable and accessible format?

Yes

Are the protocols appropriate and is the work technically sound?

Yes

Reviewer Expertise:

Bioinformatics, Genomics

I confirm that I have read this submission and believe that I have an appropriate level of expertise to confirm that it is of an acceptable scientific standard.

# Reviewer report: Reviewer response for version 1

**Published:** 2025-03-07

**Authors:** Jose Martinez

**Affiliations:** 1 Division of Plant Industry/Entomology, Florida Department of Agriculture and Consumer Services, Gainesville, Florida, USA

The dataset is designed with a well-organized and systematic approach, complemented by clear and accessible descriptions. The carefully chosen protocols and methodologies ensure both precision and reproducibility. With standardized procedures and enriched with detailed illustrations, the dataset is easily accessible and serves as an invaluable resource for a wide array of research applications.

 Additionally, the work of Davis and Griffith has resulted in a comprehensive and meticulously assembled genome of the True Lover's Knot moth, *Lycophotia porphyrea* (Denis & Schiffermüller), derived from sequencing a single male specimen. This assembly, which boasts an exceptionally high BUSCO score, is organized into 31 chromosomal pseudomolecules, including a fully reconstructed Z chromosome. The genome, created through careful methodological rigor, provides a detailed and high-quality genomic resource.

 The authors have thoroughly detailed the biological and ecological history of this species and highlighted concerns regarding its decline in certain regions of Europe. As such, this genomic resource has the potential to offer valuable insights into understanding this phenomenon.

Are sufficient details of methods and materials provided to allow replication by others?

Yes

Is the rationale for creating the dataset(s) clearly described?

Yes

Are the datasets clearly presented in a useable and accessible format?

Yes

Are the protocols appropriate and is the work technically sound?

Yes

Reviewer Expertise:

Evolutionary Biology, Ecology, IPM, Integrative Biology, Entomology, Genomics

I confirm that I have read this submission and believe that I have an appropriate level of expertise to confirm that it is of an acceptable scientific standard.

# Reviewer report: Reviewer response for version 1

**DOI:** 10.21956/wellcomeopenres.25213.r104930

**Published:** 2024-10-18

**Authors:** Arun Arumugaperumal

**Affiliations:** 1 Department of Biotechnology, Rajalakshmi Engineering College, Thandalam 602105, Chennai, Tamil Nadu, India

The first author's affiliation 'Independent researcher, Lanarck, Scotland, UK' does not provide adequate information. The author could not be identified even in near future with this very short information. The author should provide his full residential address in case not affiliated with any academic institution. Otherwise an ORCID is sufficient. The author will be associated with his past and future scientific contributions in that way.

 The already published article by an author of the same name carries a slightly different affiliation.[1]

 This is the first report of the genome sequence of *Lycophotia porphyrea* commonly known as the True Lover's Knot. There is a rope-knot with the same name. We are living in the world of Generative AI. So it is better to describe it as the True Lover's Knot moth. 

 The genome has been sequenced using long-read technology and improved further using Hi-C data. The final assembly was of size 542 Mb. The assembly has a completeness of 98.8%

 In the reference list,

 Wellcome Sanger Institute: The genome sequence of the True Lover's Knot moth, Lycophotia porphyrea (Denis & Schiffermüller), 1775. European Nucleotide Archive, [dataset], accession number PRJEB61365, 2024.

 For the above reference the ENA dataset was not found to have the exact title provided. Kindly modify it and provide a URL link to the dataset. Is this article citing itself here? Please avoid this.

Are sufficient details of methods and materials provided to allow replication by others?

Yes

Is the rationale for creating the dataset(s) clearly described?

Yes

Are the datasets clearly presented in a useable and accessible format?

Yes

Are the protocols appropriate and is the work technically sound?

Yes

Reviewer Expertise:

Bioinformatics, Genomics

I confirm that I have read this submission and believe that I have an appropriate level of expertise to confirm that it is of an acceptable scientific standard, however I have significant reservations, as outlined above.

## References

[1] : The genome sequence of the Purple Clay moth, Diarsia brunnea (Denis & Schiffermüller) 1775. *Wellcome Open Research* .2024;9: 10.12688/wellcomeopenres.22868.1 10.12688/wellcomeopenres.22868.1 PMC1141355739309223
